# Beta drives brain beats

**DOI:** 10.3389/fnsys.2014.00155

**Published:** 2014-08-27

**Authors:** Sundeep Teki

**Affiliations:** ^1^Auditory Cognition Group, Wellcome Trust Centre for Neuroimaging, University College LondonLondon, UK; ^2^Institute of Neuroscience, Newcastle UniversityNewcastle-upon-Tyne, UK

**Keywords:** interval timing, beta oscillations, timing and time perception, sensorimotor integration, beat perception, beat-based timing, interval tuning

Beta-band oscillations in basal ganglia-cortical loops have been shown to be linked to motor function in both healthy and pathological states (Brown, 2007; Engel and Fries, 2010). As well as coordinating motor activity, for instance when we synchronize our movements to a beat, the basal ganglia including the putamen and the caudate are said to contain specialized timekeeping mechanisms for perceptual timing (Buhusi and Meck, 2005). However, the exact neurophysiological role of beta-band oscillatory activity in the striatum in interval timing is not yet known.

In a recent study published in *The Journal of Neuroscience*, Bartolo et al. (2014) examined the role of beta oscillations in interval timing by directly recording from the putamen in macaques during a rhythmic synchronization task. In this paradigm, a metronome is used to establish an isochronous beat (synchronization phase) and the variability in the subsequent taps produced by the subject after the metronome is turned off (continuation phase) is evaluated as an index of internal timing behavior. Single-unit as well as local field potential (LFP) activity in the putamen in the beta and gamma range was analyzed to determine the neural correlates of interval timing.

They found that LFPs in the beta band exhibit interval tuning and showed a preference for intervals with duration around 800 ms, similar to the preferred duration observed in medial premotor cortex neurons (Merchant et al., 2013). They also reported tuning for serial order (or the task phases): LFPs in the beta band showed preferential tuning for the continuation phase that relies on internal timing whilst gamma band LFPs showed a bias toward the synchronization phase that depends on precise sensory coding. Another significant result suggests that beta oscillations are coherent at large electrode distances within the putamen that may reflect functional coordination across several areas of the putamen, and possibly the wider sensorimotor network for temporal processing. On the other hand, gamma oscillations showed small coherence associated most likely with local stimulus processing.

The present study and previous work from this group (Merchant et al., 2011, 2013) are based on an arduous methodological approach where macaques are trained to perform complex tapping tasks based on sequences of intervals rather than single isolated intervals and disambiguating stimulus-related neural activity from movement-related responses (Perez et al., 2013). Natural acoustic signals consist of sequences of time intervals, and it is important not only to encode the duration of individual sounds but also the serial order in which they are presented. The current study is the first to show evidence of both interval and serial order tuning in such sequences in the beta-band responses in the putamen.

The present study only addresses internal timing in regular sequences and it is not yet known how temporal jitter would affect the putaminal beta-band activity. In irregular sequences, beta-band modulation may remain the same or change as a function of the amount of jitter. As natural sounds are rarely presented in a temporally regular context, future work needs to address how the brain perceives time in irregular sequences. Related to this question, Fujioka et al. (2012) used magnetoencephalography to examine the nature of beta-band responses in the auditory cortex during passive listening to regular and random sequences. They found that the beta desynchronization after stimulus onset did not vary with the regularity of the stimulus, but the subsequent beta rebound peaked just before the next sound onset only for the regular stimuli.

Temporal processing in irregular sequences has been shown to be preferentially mediated by a network based in the cerebellum whilst timing in regular sequences is carried out by a striato-frontal network (Teki et al., 2011). The cerebellum is another key node of the temporal processing network (Ivry and Spencer, 2004) and recent theoretical models emphasize the involvement of both the cerebellum and the basal ganglia that are inter-connected with each other and the cortex through multiple synaptic pathways (Teki et al., 2012; Allman et al., 2014). Fujioka et al. (2012) demonstrated beta-band coherence between auditory and motor-related areas including the cerebellum but it remains to be investigated whether such functional coupling during interval timing is driven by the cerebellum, the basal ganglia, or by a different brain area that influences activity in both these core timing areas.

Bartolo et al. (2014) only briefly mention predictive coding mechanisms in bottom-up (synchronization phase) and top-down (continuation phase) processing. Bastos et al. (2012) recently proposed a canonical microcircuit for predictive coding based on iterative message passing between bottom-up stimulus-driven processing (in the gamma range) by superficial cortical neurons and top-down coding of predictive signals by deep cortical cells (in the beta range). A predictive model can be invoked in the context of the present rhythmic synchronization task where tapping to the metronome during the synchronization phase helps establish an internal model of the timing between successive metronome pulses and subsequent taps following the removal of the metronome may be driven by predictive signals. The findings of preferential gamma modulation during the synchronization phase and beta modulation during the continuation phase ties in with such a predictive coding framework. A hierarchical model for predictive coding may be anatomically realizable even locally within the striatum where there is evidence of prediction error coding by the caudate nucleus and ventral striatum whilst the putamen encodes the prediction (Haruno and Kawato, 2006). This hypothesis is supported by functional magnetic resonance imaging data showing that the putamen is specifically involved in internal prediction of beats rather than detection of beats (Grahn and Rowe, 2013).

In summary, this stimulating work on the role of putamen during internally driven timing behavior significantly adds to previous work by Merchant and colleagues based on recordings from medial premotor cortex (Merchant et al., 2011, 2013). It emphasizes the role of endogenous beta oscillations in the basal ganglia as a principal feature of interval timing in a regular, beat-based context. The study underlines the importance of local electrophysiological recordings in key areas of the animal brain to inform models of interval timing and rhythmic synchronization in humans (Teki et al., 2012) as well as non-human primates (Merchant and Honing, 2014).

## Conflict of interest statement

The author declares that the research was conducted in the absence of any commercial or financial relationships that could be construed as a potential conflict of interest.

## References

[B1] Allman M. Teki S. Griffiths T. D. Meck W. H. (2014). Properties of the internal clock: first- and second-order principles of subjective time. Ann. Rev. Psychol. 65, 743–771 10.1146/annurev-psych-010213-115117 24050187

[B2] Bartolo R. Prado L. Merchant H. (2014). Information processing in the primate basal ganglia during sensory-guided and internally driven rhythmic tapping. J. Neurosci. 34, 3910–3923 10.1523/JNEUROSCI.2679-13.2014 24623769 PMC6705277

[B3] Bastos A. M. Usrey W. M. Adams R. A. Mangun G. R. Fries P. Friston K. J. (2012). Canonical microcircuits for predictive coding. Neuron 76, 695–711 10.1016/j.neuron.2012.10.038 23177956 PMC3777738

[B4] Brown P. (2007). Abnormal oscillatory synchronization in the motor system leads to motor impairment. Curr. Opin. Neurobiol. 17, 656–664 10.1016/j.conb.2007.12.001 18221864

[B5] Buhusi C. V. Meck W. H. (2005). What makes us tick? Functional and neural mechanisms of interval timing. Nat. Rev. Neurosci. 6, 755–765 10.1038/nrn1764 16163383

[B6] Engel A. Fries P. (2010). Beta-band oscillations – signalling the status quo? Curr. Opin. Neurobiol. 20, 156–165 10.1016/j.conb.2010.02.015 20359884

[B7] Fujioka T. Trainor L. J. Large E. W. Ross B. (2012). Internalized timing of isochronous sounds is represented in neuromagnetic beta oscillations. J. Neurosci. 32, 1791–1802 10.1523/JNEUROSCI.4107-11.2012 22302818 PMC6703342

[B8] Grahn J. A. Rowe J. B. (2013). Finding and feeling the musical beat: striatal dissociations between detection and prediction of temporal regularity. Cereb. Cortex 23, 913–921 10.1093/cercor/bhs083 22499797 PMC3593578

[B9] Haruno M. Kawato M. (2006). Different neural correlates of reward expectation and reward expectation error in the putamen and caudate nucleus during stimulus-action-reward association learning. J. Neurophysiol. 95, 948–959 10.1152/jn.00382.2005 16192338

[B9a] Ivry R. B. Spencer R. M. (2004). The neural representation of time. Curr. Opin. Neurobiol. 14, 225–232 10.1016/j.conb.2004.03.013 15082329

[B11] Merchant H. Honing H. (2014). Are non-human primates capable of rhythmic entrainment? Evidence for the gradual audiomotor evolution hypothesis. Front. Neurosci. 7:274 10.3389/fnins.2013.00274 24478618 PMC3894452

[B12] Merchant H. Perez O. Zarco W. Gamez J. (2013). Interval tuning in the primate medial premotor cortex as a general timing mechanism. J. Neurosci. 33, 9082–9096 10.1523/JNEUROSCI.5513-12.2013 23699519 PMC6705035

[B13] Merchant H. Zarco W. Perez O. Prado L. Bartolo R. (2011). Measuring time with different neural chronometers during a synchronization-continuation task. Proc. Natl. Acad. Sci. U.S.A. 108, 19784–19789 10.1073/pnas.1112933108 22106292 PMC3241773

[B14] Perez O. Kass R. E. Merchant H. (2013). Trial time warping to discriminate stimulus-related from movement-related neural activity. J. Neurosci. Methods 212, 203–210 10.1016/j.jneumeth.2012.10.019 23147009

[B15] Teki S. Grube M. Griffiths T. D. (2012). A unified model of time perception accounts for duration-based and beat-based timing mechanisms. Front. Integr. Neurosci. 5:90 10.3389/fnint.2011.00090 22319477 PMC3249611

[B16] Teki S. Grube M. Kumar S. Griffiths T. D. (2011). Distinct neural substrates of duration-based and beat-based auditory timing. J. Neurosci. 31, 164–171 10.1523/JNEUROSCI.3788-10.2011 21389235 PMC3074096

